# Supplementary material for: Resolving the controls of water vapour isotopes in the Atlantic sector
Source: Nat Commun. 2019 Apr 9;10:1632. doi: 10.1038/s41467-019-09242-6 (PMC6456600; doi:10.1038/s41467-019-09242-6)
Supplement: Supplementary file 1 — Supplementary Information [file 41467_2019_9242_MOESM1_ESM.docx]

**Supplementary information for**

**Resolving the controls of water vapour isotopes in the Atlantic sector**

Bonne et al.


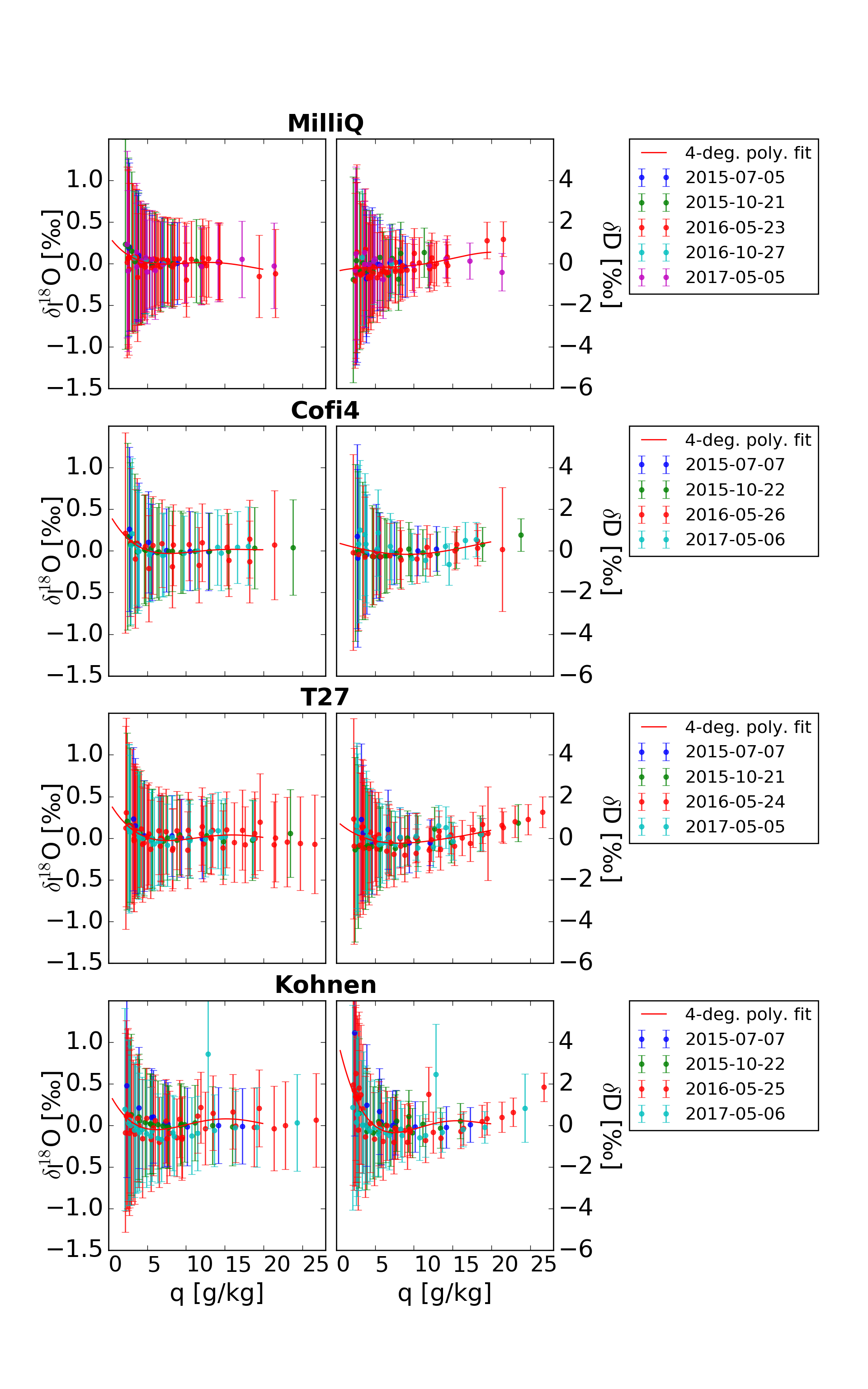


**Supplementary Figure 1:** **Humidity response functions experiments for the water vapour isotopic analyser.** Humidity response functions experiments are presented for the four used isotopic standards (from top to bottom, standards named MilliQ, Cofi4, T27 and Kohnen) expressed as a deviation from the mean isotopic value between 10 000 and 25 000 ppm. Measured values are displayed with dots as the mean value and vertical error bars representing the standard deviation of the isotopic value during the time of the injection. Experiments conducted in July 2015, October 2015, May 2016 and May 2017 are respectively presented in blue, green, red and cyan. The calculated response functions are displayed for the range of encountered ambient air humidity values in plain red lines.

**
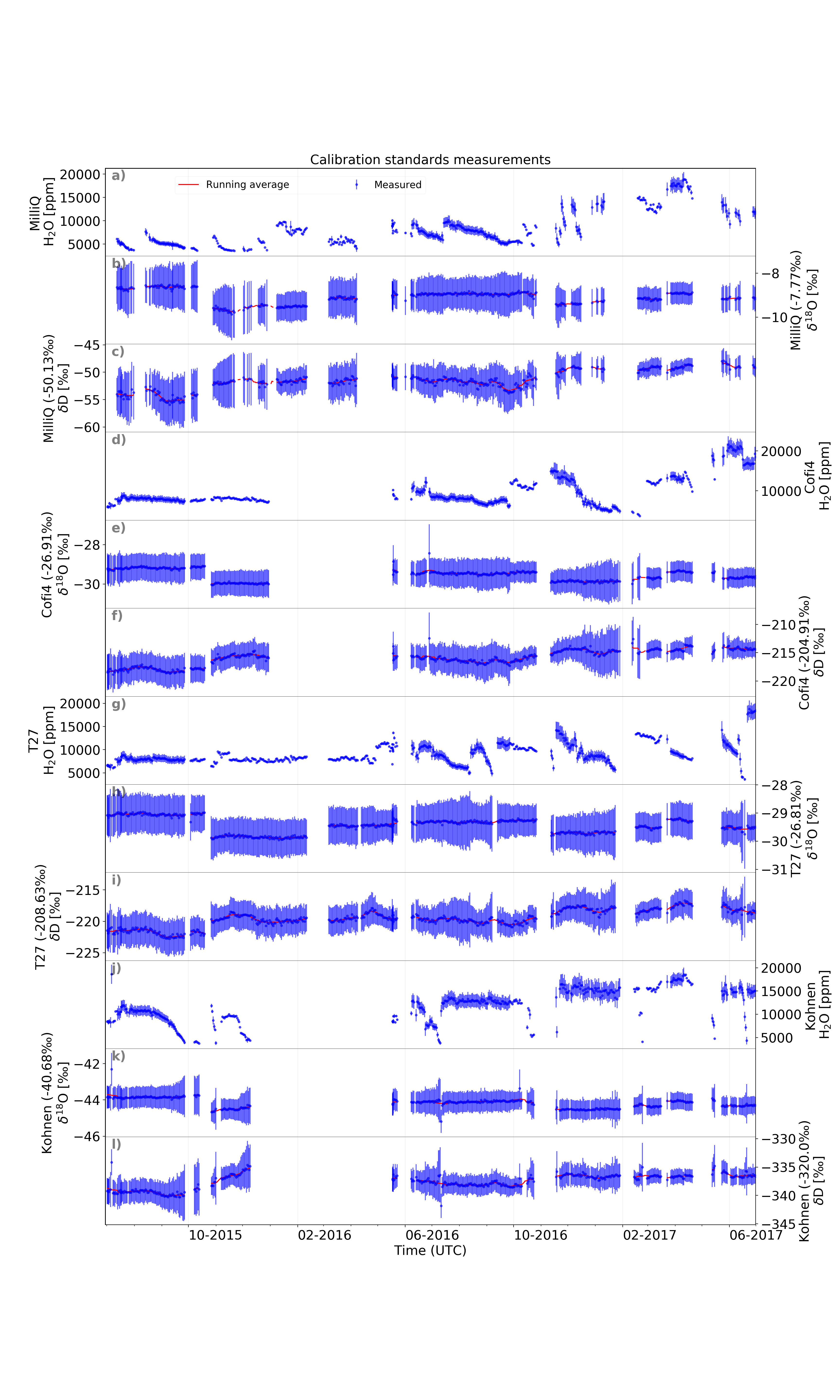
**

**Supplementary Figure 2: Measured isotopic values of the different calibration standards during the period 2015-06-29 to 2017-06-30.** From top to bottom, the measurements of all different standards (named MilliQ, Cofi4, T27 and Kohnen) are successively displayed for the humidity level (in ppm), the $\delta^{18}O$ and the $\delta^{2}H$ corrected from humidity (in ‰). Blue dots represent the mean and blue error bars represent standard deviation of the last 15 minutes of each 30-minute standard measurement. Outliers, which can be caused by instabilities of injections by the calibration system, have been removed from these data series. The red curves represent the 14-days running averages of these measurements, used for the computation of the calibration curves. The expected isotopic composition of each standard, as determined by independent laboratory measurements are given for each standard in the y-labels for $\delta^{18}O$ and the $\delta^{2}H$.

**a)**


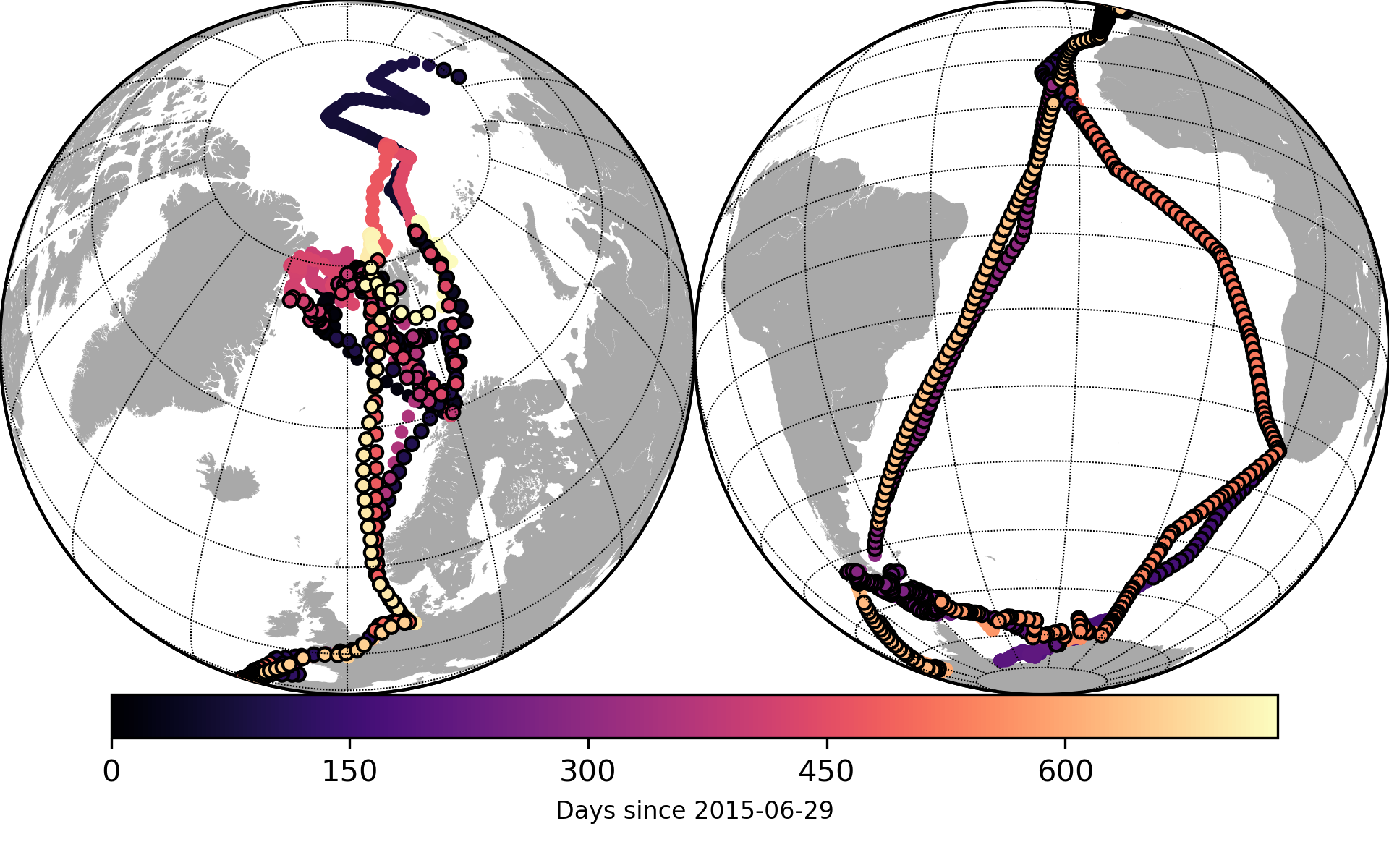


**b)**

**
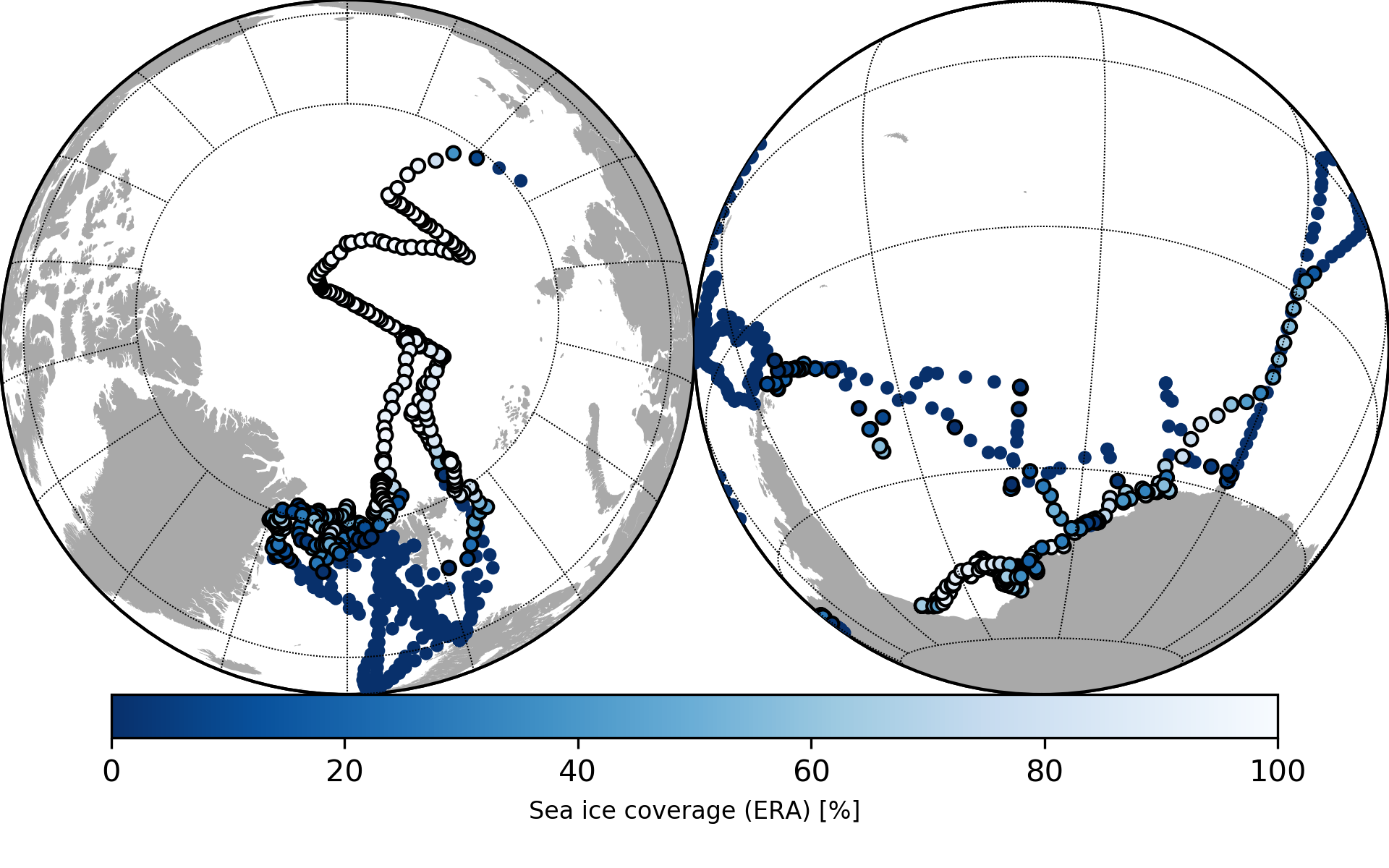
**

**Supplementary Figure 3:** **Locations of vapour isotopic measurements on-board Polarstern, local sea ice cover and filtered areas at open-ocean and within sea ice.** Each dot indicates the location of an available observation of vapour isotopic composition at 6-hours resolution between 2015-06-29 and 2017-06-30. a) Colours indicate the time passed since measurement start at 2015-06-29. Data points for unbiased open-ocean areas are drawn with a black edge (see Methods for details on data filtering). b) Colours indicate the local sea ice coverage around Polarstern. Data points with local sea ice coverage above 0 % are drawn with a black edge (see Methods for details on data filtering).

**Supplementary Figure 4: Independence of d-excess regarding wind speed under different** $\mathbf{RH}_{\mathbf{sea}}$ **and SST regimes.** Observed d-excess values are displayed as blue dots as a function of the wind speed for different ranges of $\mathrm{RH}_{\mathrm{sea}}$ and SST. All horizontally (vertically) distributed subplots have the same ranges of SST values ($\mathrm{RH}_{\mathrm{sea}}$ values). From left to right, $\mathrm{RH}_{\mathrm{sea}}$ ranges are successively: 50 - 60%, 60 - 70%, 70 - 80%, 80 - 90%, 90 - 100% and 100 - 125%. From bottom to top, SST ranges are: -1.8 to 5°C, 5 to 10°C, 10 to 15°C, 15 to 20°C, 20 to 25°C and 25 to 30°C. Vertical red lines represent the 7 m s^-1^ wind speed threshold.

**Supplementary Figure 5:** **Comparison of observations with closure assumption calculations.** Time series of measurements and calculations based on the closure assumption during the period 2015-06-29 to 2017-06-30 at 6-hours resolution, over open ocean, only. (a) $\delta^{18}O$ (indigo) and d-excess (cyan) of surface oceanic water. (b) Measured air relative humidity (purple) and calculated sea surface relative humidity (red), see Method section for details. (c) Measured air temperature (indigo), sea surface temperature (red), and specific humidity (light blue). (d) Measured vapour $\delta^{18}O$ values (light blue), and calculated $\delta^{18}O$ based on the closure assumption for MJ79_ref_ (dark red). (e) Measured vapour d-excess values (light blue), and calculated d-excess values based on the closure assumption for MJ79_ref_ (dark red). (f) Polarstern latitude (light blue) and sea ice coverage (red). (g) Wind speed (light blue) and 7 m s^-1^ threshold value to distinguish between a smooth and rough wind regime (grey horizontal line).

**Supplementary Figure 6:** **d-excess values from observations, closure assumption calculations and isoGCM simulations during a south-north Atlantic transect.** Time series of d-excess measurements (thick blue lines) compared to theoretical calculations based on the closure assumption (upper plot) and isoGCM simulation results (lower plot), for the period 2016-04-10 to 2016-05-11 during an Atlantic North-South transect from Bremerhaven (Germany) to Cape Town (South Africa) over open ocean, only. For the closure assumption, two calculations with kinetic fractionation coefficients related to smooth and to rough wind regimes are respectively displayed in light green and dark green (see Methods section). For the isoGCM results, both outputs from ECHAM_final_ and ECHAM_exp_ simulations are compared, respectively displayed in dark blue and orange.

# ****

**Supplementary Figure 7: Evaluation of the closure assumption calculations of d-excess against observations.** Scatter plot of measured versus calculated d-excess values (in blue) for the period 2015-06-29 to 2017-06-30 at 6-hours resolution over open ocean, only. For the calculations based on the closure assumption, three different types of wind speed dependency are assumed (see text for details): (a) wind speed dependent fractionation coefficients for smooth and rough wind regimes MJ79_ref_, (b) a constant fractionation coefficient for smooth wind regime conditions MJ79_smooth_ and (c) a constant fractionation coefficient for rough wind regime conditions MJ79_rough_. The associated linear regressions are displayed and the corresponding equations are given in red. Black lines represent the 1:1 slopes.

1. (b)

**Supplementary Figure 8: Evaluation of the isoGCM simulated d-excess against observations.** Scatter plots of measured versus simulated d-excess values during the period 2015-06-29 to 2017-06-30 at 6-hours resolution, for two different isoGCM simulations (see text for details): ECHAM_exp_ (orange) and ECHAM_final_ (blue). (a) Measured and simulated d-excess values for open ocean, only. (b) Measured and simulated d-excess values for sea ice covered areas, only. Associated linear regressions and the corresponding equations are given in both plots with the same colours as the datasets. Black lines represent the 1:1 slopes.

**Supplementary Figure 9:** **Variations of measured isotope values,** $\mathbf{RH}_{\mathbf{sea}}$ **and SST with sea ice coverage.** Scatter plots (blue) of measurements versus local sea ice coverage for (a) $\delta^{18}O$, (b) d-excess, (c) SST, (d) $T_{\mathrm{air}}$, (e) Northern hemisphere latitudes, (f) Southern hemisphere latitudes, (g) $\mathrm{RH}_{\mathrm{sea}}$,. Associated linear regressions and the corresponding equations are given in red in all plots. Data for sea ice coverage higher than 0 % are shown for the period 2015-06-29 to 2017-06-30.

**Supplementary Figure 10**: **Observed and isoGCM-simulated d-excess and sea ice coverage during an Arctic expedition.** Time series for the period 2015-08-15 to 2015-09-30 in the Arctic sector of d-excess (upper plot) and sea ice coverage (lower plot). Observations from Polarstern are shown with thick light blue lines. IsoGCM simulation results are added for comparison, assuming either a bare (ECHAM_exp_, orange line) or a snow-covered (ECHAM_final_, dark blue line) sea ice surface (see Methods for details). Gold shading indicates the locations with sea ice coverage higher than 0 %.

| **Dataset number** | **Dataset description** | **Filtration applied** | **Operation applied** |
| --- | --- | --- | --- |
| 1 | **1 s data of 30 min standard measurements** |  |  |
| 2 | Averages of the  last 15 min of standard measurements |  | 15 min averages of dataset #1 |
| 3 | Selected corrected averaged values of all measurements of every standards | 1) Manual flags  2) 5000 ppm < $\bar{H_{2}O}$ < 28000 ppm  3) $\sigma(H_{2}O)$ < 2500 ppm  4) ${\sigma(\delta}^{18}O)$ < 1.5 ‰  5) ${\sigma(\delta}^{2}H)$< 5 ‰ | 1) Humidity concentration dependence correction  2) Isotopic correction to the reference line known valued |
| 4 | Running average | At least 3 values | 14 days running average of dataset #3 |
|  |  | Values before and after within 7 days |  |
|  |  | no restart between previous and next values |  |
| 5 | Distance to running average |  | Difference between datasets #4 and #3 |
| 6 | Selected corrected averaged values of all measurements of every standards | Distance to running average below 1.5, 5 and 8 ‰ for $\delta^{18}O$, $\delta^{2}H$ and d-excess |  |
| 7 | **Daily values of running averages for all standards to compute calibration curves** | 1) At least 3 values  2) Values before and after within 7 days  3) No restart between previous and next values | 14 days running average of dataset #6 |

**Supplementary Table 1: Filtration and correction steps applied for the computation of 14-days running averages of the liquid standard measurements used for the calculation of the calibration curves.**
